# Size Matters: A Computational Study of Hydrogen Absorption in Ionic Liquids

**DOI:** 10.1021/acs.jcim.3c01688

**Published:** 2023-12-21

**Authors:** Alejandro Rivera-Pousa, Raúl Lois-Cuns, Martín Otero-Lema, Hadrián Montes-Campos, Trinidad Méndez-Morales, Luis Miguel Varela

**Affiliations:** † Grupo de Nanomateriais, Fotónica e Materia Branda, Departamento de Física de Partículas, Universidade de Santiago de Compostela, Campus Vida s/n, Santiago de Compostela E-15782, Spain; ‡ Instituto de Materiais (iMATUS), Universidade de Santiago de Compostela, Avenida do Mestre Mateo 25, Santiago de Compostela E-15782, Spain; § CIQUP, Institute of Molecular Sciences (IMS)—Departamento de Química e Bioquímica, Faculdade de Ciências da Universidade do Porto, Rua Campo Alegre, Porto 4169-007, Portugal

## Abstract

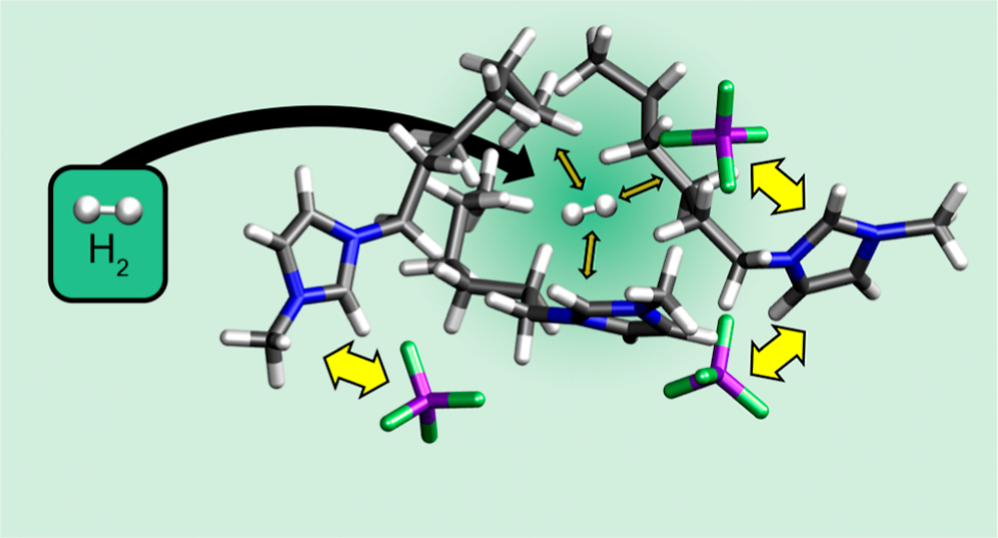

We combined both density functional theory and classical molecular dynamics simulations to investigate the molecular mechanisms governing hydrogen solvation in a total of 12 ionic liquids. Overall, the analysis of the structural properties under high temperature and pressure conditions revealed weak interactions between hydrogen and the ionic liquids, with a slight preference of this gas to be placed at the apolar domains. Interestingly, those ionic liquids comprising nitrate anions allow the accommodation of hydrogen molecules also in the polar areas. The study of the hydrogen velocity autocorrelation functions supports this observation. In addition, the structure of all of the tested ionic liquids was almost insensitive to the addition of hydrogen, so the available free volume and cavity formation are presumably the most important factors affecting solubility.

## Introduction

Energy transition to prevent climate change is a major priority worldwide. Therefore, many research efforts have been devoted to the development and improvement of a new energy model that meets the needs of a growing population while supporting low-carbon growth. For instance, carbon capture techniques have attracted much attention of the academia and industry as a viable option to decrease greenhouse gas emissions.^1^ Moreover, the increasing demand for sustainable energy has led to the investigation of alternative sources such as solar, wind, or nuclear energy.

In this context, hydrogen (H_2_) can be considered as a clean and promising energy vector to substitute fossil fuels. However, a critical aspect of the “hydrogen economy” that must be taken into account for its general implementation is the development of efficient storage mechanisms at low pressures. To answer this challenge, a large number of hydrogen storage techniques are being investigated as a long-term solution, including both materials-based physical storage and materials-based chemical storage. In the first case, these media include porous materials such as zeolites,^2^ clathrates,^3^ metal–organic frameworks,^4^ or porous liquids,^5^ and the adsorption capacity mainly depends on the specific surface area, the pore size, and the working pressure and temperature. On the other hand, the main idea behind chemical storage is to use hydrogen carriers to design materials that can undergo hydrogenation and dehydrogenation processes. A large number of systems can be attractive for the latter purpose, e.g., the potential ability of borohydrides^6^ and ammonia borane^7^ to store and deliver large amounts of molecular hydrogen has been intensively investigated. Such dehydrogenation reactions were found to be enhanced in the presence of ionic liquids (ILs),^8−15^ both in terms of the extent and rate of hydrogen release.

ILs are salts composed of both organic and inorganic cations and anions that become liquid around or below 100 °C. Tailoring the ion structures makes it potentially possible to design the IL with the required properties for almost any application, including low viscosity to overcome limitations related to mass transfer, or good thermal and chemical stability to endure hydrogenation/dehydrogenation conditions, among others.^16−20^ Under these circumstances, having reliable solubility data of gases in ILs is very important not only for the design and operation of chemical reactions but also to use ILs as a gas-separation medium or in extraction systems.^21−24^ Therefore, factors affecting the solubility have been thoroughly evaluated in the past few years by means of experimental measurements and computational modeling.

From the experimental point of view, much attention has been devoted to understanding the influence of the temperature and pressure on gas solubility in ILs. However, the case of H_2_ sorption in ILs is controversial since different sources reported conflicting data sets with opposite temperature behaviors. For example, some authors identified a higher solubility with rising temperature,^25−32^ which is an inverse trend to that of the vast majority of gases in ILs but can be very useful in applications such as gas separation. On the other hand, lower hydrogen solubility with increasing temperature has also been reported,^33−37^ and even measurements exhibiting a maximum close to room temperature and at 0.1 MPa can be found in the literature.^38,39^ These inconsistencies were attributed to the difficulties of accurately measuring such a low-solubility gas. Indeed, Brennecke and co-workers^40^ were not able to measure its solubility in 1-butyl-3-methylimidazolium hexafluorophosphate ([BMIM][PF_6_]) at 25 °C since it was below their experimental resolution.

Theoretical methods are very useful to reduce experimental difficulties and costs when screening a large number of compounds. In addition, they can also offer an estimation of the solubility limit and provide fundamental knowledge about the molecular mechanism for the dissolution of gas molecules in ILs. Thus, researchers have, for example, employed COSMO-based methods,^41,42^ Monte Carlo (MC) simulations,^43−46^ and Widom test-particle insertion (TPI)^47^ or grand canonical MC (GCMC)^48^ approaches to analyze the solubility of molecular hydrogen in different ILs and its dependence with factors such as temperature or molar volume. Moreover, molecular dynamics (MD) simulations have been previously carried out to study the molecular diffusion of binary mixtures of an IL with dissolved gases,^45,49,50^ and larger diffusivities for hydrogen compared with those of all the gases other than helium were reported. On the other hand, works based on density functional theory (DFT) calculations were also used to obtain an accurate description of the interaction of the IL ions (mainly the anion) with H_2_.^51−53^

However, despite its well-known utility in gas separation processes, computational data about mixtures of molecular H_2_ and ILs remain scarce in comparison with those of other gases, such as CO_2_. Thus, the aim of this work is to obtain a more detailed understanding of the microscopic factors regulating hydrogen solubility in ILs. This can be achieved through the analysis of the interaction mechanism between H_2_ and the ions of the IL and also by the analysis of the tendency of ILs to generate interstitial void space in the bulk due to anion–cation interactions and to the tendency to segregate into polar and apolar domains.

**Figure 1:**
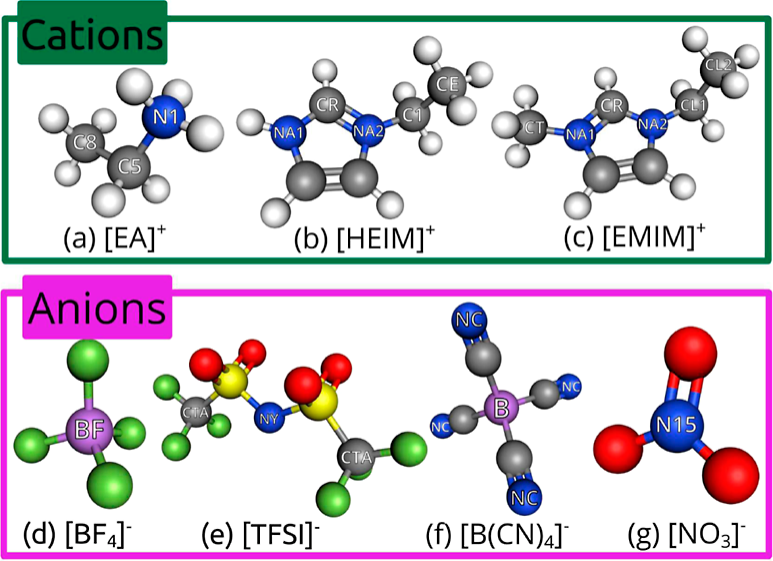
3D models of the ions in the simulations. Gray, blue, white, green, violet, red, and yellow correspond to carbon, nitrogen, hydrogen, fluorine, boron, oxygen, and sulfur, respectively.

For that purpose, we performed several MD and DFT calculations in order to study the solvation environments of H_2_ molecules in several ILs, which were chosen with the objective of screening a wide variety of ion combinations that are representative of the most used protic and aprotic ILs. The studied IL pairs were ethylammonium nitrate ([EA][NO_3_]), 1-ethylimidazolium nitrate ([HEIM][NO_3_]), 1-ethylimidazolium bis(trifluoromethanesulfonyl)imide ([HEIM][TFSI]), 1-ethyl-3-methylimidazolium tetracyanoborate ([EMIM][B(CN)_4_]), 1-ethyl-3-methylimidazolium bis(trifluoromethanesulfonyl)imide ([EMIM][TFSI]), 1-ethyl-3-methylimidazolium tetrafluoroborate ([EMIM][BF_4_]), and 1-ethyl-3-methylimidazolium nitrate ([EMIM][NO_3_]). These molecular structures are shown in Figure 1 along with the atoms used for the calculations. Moreover, since the IL cations also play an important role in the solubility of H_2_, various [C_*n*_MIM][BF_4_] systems were analyzed, with *n* = {2, 4, 6, 8, 10, 12}. Thus, the  cation is the same as [EMIM]^+^, and both names will be used interchangeably throughout the text.

The outline of this work is as follows. In the Methodology section, we provide a detailed account of the simulation methods. In the Results and Discussion section, we present and discuss the results obtained, and the concluding remarks are offered in the Conclusions section.

## Methodology

### Molecular Dynamics Methods

Atomistic MD simulations of all systems were performed using the Groningen machine for chemical simulations (GROMACS) version 2020.4.^54^ The chosen force field was the all-atom version of the optimized potential for liquid simulations (OPLS-AA).^55,56^ The topology used in the simulations for [EA]^+^ cations has been already described in ref (57). On the other hand, in the nitrate anion, the nitrogen site was assigned a partial charge of *q*_N_ = +0.794*e* and a mass of *m*_N_ = 14.00670 u, while for the oxygens, partial charges of *q*_O_ = −0.598*e* and masses of *m*_O_ = 15.99940 u were selected. Their Lennard-Jones parameters are σ_N_ = 3.496 × 10^–1^ nm, ε_N_ = 7.1128 × 10^–1^ kJ/mol, σ_O_ = 3.175 × 10^–1^ nm, and ε_O_ = 8.7864 × 10^–1^ kJ/mol, respectively, in accordance with previous work done by the authors.^58^ The parameterization of the  anion was the one developed by Koller et al.,^59^ whereas the remaining topologies of the IL ions were taken from ref (60). Unscaled charges were used for the ions instead of scaled charges, which are usually used to prevent sluggish dynamics,^61,62^ since the focus of this work is the static properties and configurations. This will limit the quantitative analysis of correlation functions, but it will not affect the configurational analysis to which we will stick to. Finally, hydrogen molecules were modeled as two charged real atoms constrained by a rigid bond of 74.14 pm in length and a charged virtual Lennard-Jones site to properly mimic the quadrupole moment of the molecule. Real atoms have masses of 1.008 u and charges of +0.615*e*, while the charge of the virtual site is −1.23*e* and its Lennard-Jones parameters are σ = 2.958 × 10^–1^ nm and ε = 3.051336 × 10^–1^ kJ/mol.^63,64^

The bulk behaviors of all systems were studied in their pure forms as well as with a 5 mol % concentration of H_2_ molecules, which was chosen after a detailed solubility analysis to be discussed later in this paper. For pure ILs, the mixtures consisted of 1000 cation–anion pairs, whereas for the mixtures with H_2_, 950 cation–anion pairs were present, along with 50 H_2_ molecules.

The simulation procedure was the same for all systems. Initial configurations were created with PACKMOL.^65^ After that, energy minimization was carried out in GROMACS by using a steepest descent algorithm. A 0.1 kJ mol^–1^ nm^–1^ tolerance was used, along with an initial step of 0.01 nm. Once energy minimization had concluded, MD equilibration runs were carried out in the NPT ensemble for a total of 30 ns. Finally, 10 ns production runs in the same NPT ensemble were performed. Additional runs of 100 ps in which the velocities were saved at each step were carried out for calculations of the velocity autocorrelation functions (VACFs). In all MD simulations, a constant time step of 1 fs was employed.

Due to the low solubility of H_2_, MD simulations were performed at high pressure in order to include enough gas molecules to obtain reasonable statistics. In addition, high temperature and pressure conditions are interesting in processes such as the Fischer–Tropsch synthesis reaction for the production of hydrocarbons. Thus, the temperature was held at 550 K by means of the V-rescale thermostat^66^ using a 0.1 ps coupling constant, whereas the pressure was kept to a constant value of 50 bar by using an isotropic Parrinello–Rahman barostat with a 1 ps coupling time.^67^ As for the forces, long-range Coulomb interactions were computed using the smooth particle-mesh Ewald electrostatics method^68^ with a real space cutoff radius of 1.1 nm and a Fourier grid spacing of 0.12 nm. Finally, van der Waals forces were considered within a cutoff radius of 1.1 nm.

### Density Functional Theory Methods

In order to support the MD simulations, a series of DFT calculations were performed. The energy and geometry of the complexes formed by H_2_ with IL cations or anions were calculated by optimizing different initial configurations. All the calculations were performed using the software package Gaussian 16^69^ with the hybrid density functional ωB97X-D,^70^ which has built-in dispersion correction and was already suggested by García et al.^71^ for studying acid gas capture by ILs. We selected def2-TZVPP as the basis set.^72,73^ In addition, to take into account the basis set superposition error (BSSE), the counterpoise method was employed.^74,75^ For the optimization, the Berny algorithm was chosen, with the convergence option set to VeryTight, and Ultrafine was selected as the integration grid in the Gaussian input file, which was deemed to be sufficiently accurate for our study.

## Results and Discussion

The main results of our simulations will be analyzed below to understand the absorption process of hydrogen molecules on the IL media. The analysis is organized into three sections. The first section consists of a preliminary analysis of Hydrogen Solubility in ILs by means of Widom’s potential distribution theorem, while the second section presents the Solvation Mechanism regulating the absorption process. Finally, in the last section, the influence of the anion in H_2_ solubility is studied in more detail.

### Hydrogen Solubility

As a first step in determining the solvation of hydrogen molecules in ILs, the solubility of H_2_ in the studied systems was calculated. The solubility of a solute A in a solvent B, *s*_AB_, when both species are in equilibrium at constant temperature *T* and pressure *p* can be expressed in terms of the inverse of Henry’s constant, *k*_H_, as *s*_AB_ = *k*_H_^–1^*p*. This constant can also be related to the excess chemical potential, μ_ex,A_, as^76^
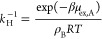
1where β = 1/*k*_B_*T* and ρ_B_ is the number density of the solvent (in our case, the number density of IL ion pairs in the system). The excess chemical potential can be computed from the results of our pure IL simulations through Widom’s potential distribution theorem.^77^ The resulting expression for the NPT ensemble^78^ is
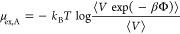
2where *V* is the volume of the simulation box and Φ is the potential energy of a gas molecule randomly inserted within the system. The brackets represent isothermal–isobaric averaging over both different insertions and solvent configurations.

For the computation of Φ, solute molecules were modeled as neutral Lennard-Jones particles with the same interaction parameters as the virtual site in the hydrogen model. It is important to note that since the most relevant multipole moment in the H_2_ molecule is quadrupolar, and on average, interactions between a quadrupole and monopoles as well as dipoles and other quadrupoles yield a negative energy contribution at high temperatures,^79^ the neglect of electrostatic interactions could result in an underestimation of the solubility. Because of this, the following results should be understood as lower bound. However, since the Lennard-Jones interaction is the dominant contribution, the electrostatic correction is expected to be relatively small. Moreover, it is important to remark that eq 2 assumes that solvent configurations are complete, i.e., that the interaction of the solvent with IL molecules does not result in arrangements that are not present in the pure system. For arbitrary solvents, this is not the case, but as it will be shown later, the weakly interacting nature of H_2_ results in no noticeable changes to the IL structure. Thus, it can be assumed that a sampling of the pure IL configurations accounts for all possible solvent arrangements in the mixtures with H_2_. A total of 10^4^ configurations were analyzed for each IL, extracted from the production runs at intervals of 1 ps. Each configuration was sampled with 10^5^ random insertions. The excess chemical potential was found to oscillate around equilibrium values during simulation time for all systems, and its time evolution is shown in Figure S1 of the Supporting Information.

The calculated solubilities are listed in Table 1. Overall, hydrogen solubility is low for all systems, in agreement with the experimental results mentioned in the Introduction section. Interestingly, when the [C_*n*_MIM][BF_4_] series is examined, it can be seen that hydrogen solubility increases as the alkyl chain in the cation grows. Moreover, the three lowest solubilities are found for [EA][NO_3_], [HEIM][NO_3_], and [EMIM][NO_3_], all of which share the same anion. Coincidentally,  is the smallest of the chosen anions, with bulkier ones showing higher solubilities. The size asymmetry seems to have a marked correlation with H_2_ solubility, and this aspect will be thoroughly studied in the following sections. Finally, regarding the distinction between protic and aprotic ILs, it can be noted that for the same choice of anion, the liquids with protic cations exhibit lower solubilities than their aprotic counterparts.

**Table 1:** Calculated Solubilities (in mole %) of H_2_ Molecules in the Studied ILs

	liquid	solubility (mol %)
protic	[EA][NO_3_]	0.136
	[HEIM][NO_3_]	0.085
	[HEIM][TFSI]	1.489
aprotic	[EMIM][B(CN)_4_]	1.783
	[EMIM][TFSI]	2.009
	[EMIM][NO_3_]	0.268
	[C_2_MIM][BF_4_]	0.946
	[C_4_MIM][BF_4_]	1.499
	[C_6_MIM][BF_4_]	2.158
	[C_8_MIM][BF_4_]	2.961
	[C_10_MIM][BF_4_]	3.834
	[C_12_MIM][BF_4_]	4.628

It is important to bear in mind that these solubility values correspond to a liquid interface in equilibrium with hydrogen gas under the specified temperature and pressure conditions. It is possible that the systems at hand could hold a higher concentration of H_2_ molecules within them, but the absorption process would not be spontaneous under the previously described conditions. Taking this into account, along with the fact that the values reported in Table 1 constitute a lower bound for the solubility due to the neglect of the electrostatic interactions, in the following sections, a molar percentage of 5 mol % for H_2_ within the ILs was chosen for the simulations.

### Solvation Mechanism

For a first insight into the structure of the mixtures at the microscopic level, radial distribution functions (RDFs) between the most representative atoms of the ILs and the center of mass of H_2_ molecules were calculated. The RDF of molecules *b* in a shell at distance *r* around a molecule *a* can be obtained from production run trajectories as
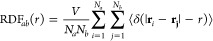
3

**Figure 2:**
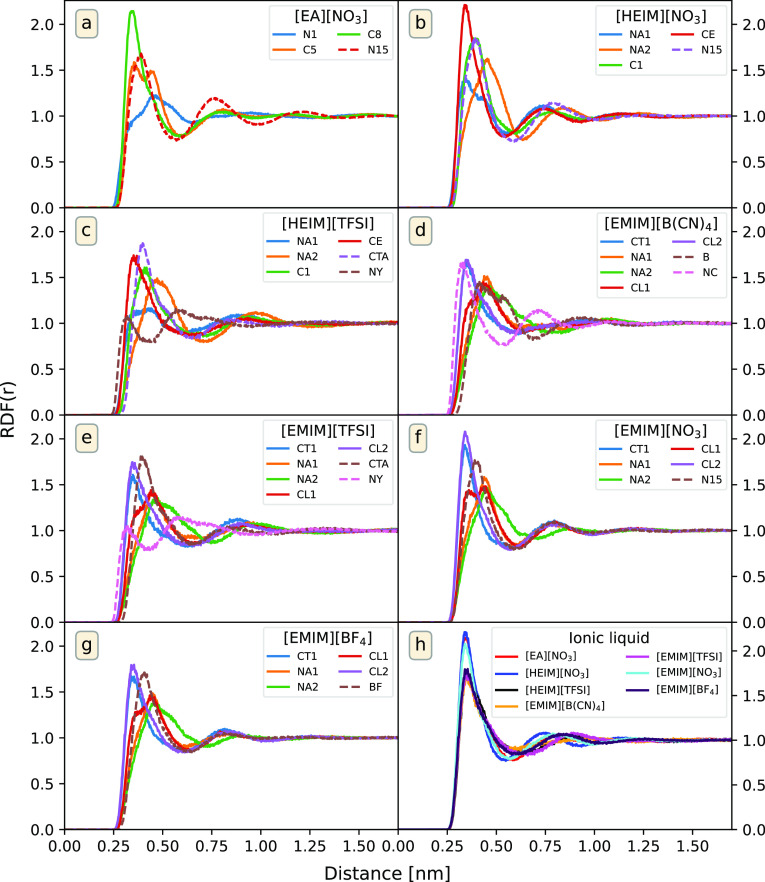
(a–g) RDFs between H_2_ molecules and various atoms from different systems. Solid lines represent the RDFs of H_2_ with respect to the atoms of the cations and dashed lines represent the ones of the anions. (h) Comparison of the RDFs between H_2_ molecules and the terminal tail atom of the cation for each system. The labels of the atoms are shown in Figure 1.

The RDFs of the studied systems, excluding the C_*n*_MIM series, are presented in Figure 2a–g, where the IL in the system is the one specified on each legend and the atoms are named following Figure 1. As can be seen in these figures, the first shell of hydrogen molecules solvating cations and anions is located at approximately the same distance. This can be seen more clearly by looking at the minimum distance distribution functions (MDDFs),^80^ which are shown in Figure S2 of the Supporting Information, where it can be seen that the primary coordination distance between gas molecules and IL molecules is practically the same for anions and cations. It is also interesting to note that the distance from the cations remains constant regardless of the system studied, H_2_ molecules being placed at approximately 0.35 nm from the nearest non-hydrogen atom. Moreover, it can also be seen how the difference in the size of the anion, similar to what was discussed when analyzing solubility, has a relevant impact on the strength of the interaction of H_2_ molecules with both cations and anions. This can be easily seen in [EMIM]^+^ systems, which exhibit higher peaks in the RDFs of the systems with smaller anions for both ions.

Regarding the cation-hydrogen RDFs, although the nature of the polar head of each cation is different, it can be seen how the behavior in the apolar areas of the molecule is similar in all cases. Moreover, it is interesting to note how for all ion pairs, the RDFs of these alkyl tails are the ones that exhibit the highest cation–H_2_ interaction. This reveals a higher density of H_2_ in the surroundings of the cation tails. The RDFs for the terminal carbon atoms of the tails (C8 for [EA]^+^, CE for [HEIM]^+^, and CL2 for [EMIM]^+^) are shown in Figure 2h to compare them straightforwardly. The figure shows how the coordination distance is the same for all systems, and the differences appear only in the heights of the peaks when the anion is changed. In particular, a stronger interaction between hydrogen and the terminal carbon atom is observed in the presence of . The influence of the anions will be analyzed in detail in the Influence of the Anion section.

**Figure 3:**
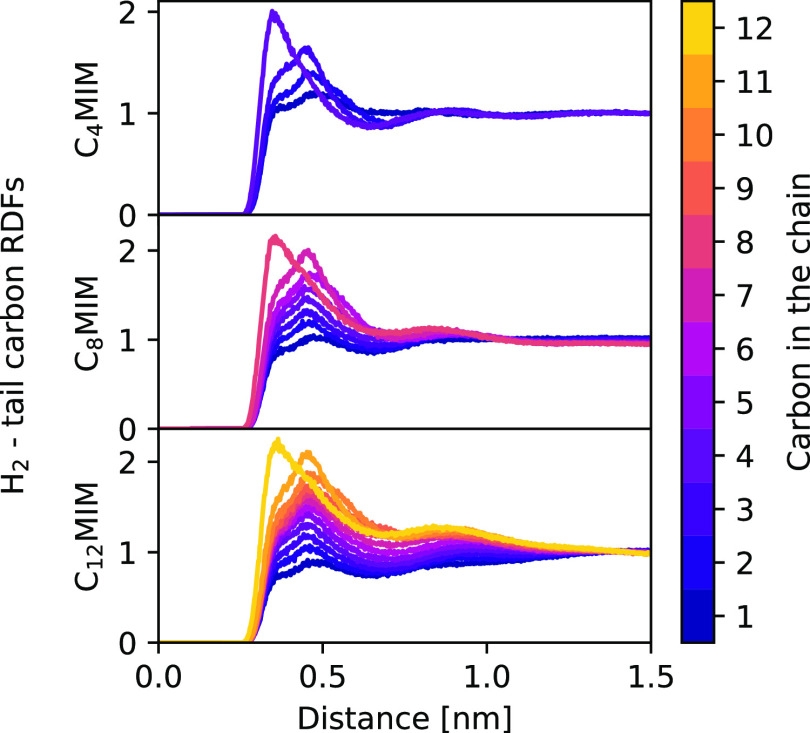
RDFs between H_2_ molecules and each carbon atom in the alkyl chain of the  cation for various systems. Carbon atoms are indexed, starting from the one closest to the imidazolium ring.

To further analyze the behavior of the gas molecules around the aliphatic tails, the RDFs between the  cations and H_2_ molecules were calculated. These RDFs are shown in Figure 3. The number on the color bar represents the position of each carbon atom in the tail, starting from the one closest to the planar cationic ring. As can be seen in that figure, the interaction with the terminal carbons is independent of the length of the alkyl tail. Moreover, a consistent structure can be observed when this length is increased. A first peak corresponding to the terminal carbon atom, where the probability of finding H_2_ molecules reaches a maximum, is followed by a secondary peak at a larger coordination distance corresponding to the next carbon atom in the chain.

From there, the remaining carbon atoms show a coordination distance similar to the previously mentioned one, with the height of the peaks decreasing as the imidazolium ring is approached. Overall, the results show a tendency of H_2_ molecules to stay away from the charged head of imidazolium-based cations and place themselves as far away as possible from the ring. This structural picture is compatible with a “solvophobic solvation” of molecular hydrogen in ILs, which is expected to increase with the cation alkyl chain length. This solvophobic solvation is analogous to the hydrophobic effect in aqueous solutions, which has been studied for solutes with both nonpolar and polar character.^81,82^ In particular, it has been shown that solubilities of gases such as H_2_ in clay interlayer water are consistent with hydrophobic solvation theory.^83^ This result can be further confirmed by looking at the MDDFs between hydrogen molecules and IL ions. These functions are shown in Figure S3 of the Supporting Information. It can be seen that the MDDF between cations and hydrogen molecules does not change with the length of the alkyl chain, which is consistent with H_2_ being located at the same position relative to the tails near the terminal carbon atom. Moreover, the coordination distance between H_2_ and anions is also left unchanged when the length of the aliphatic tail is increased. From all these results, it is easy to assume that gas molecules do not interact to a large extent with the ions and, therefore, the solvation mechanism is not directly related to interactions between the gas molecules and the IL. Similarly, weak RDFs between H_2_ and ILs can be found in the literature for different combinations of anions and cations.^48^

**Figure 4:**
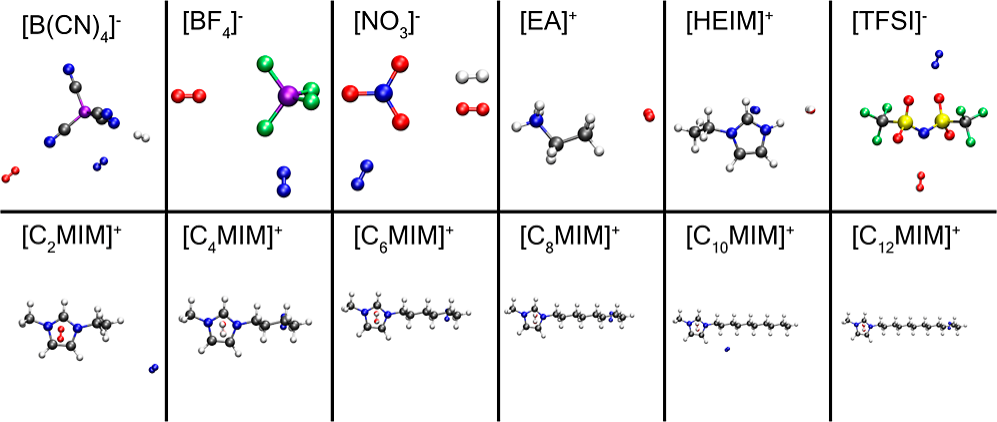
Snapshots of the three final configurations with the lowest binding energies for the different ion–H_2_ systems. The color of the H_2_ molecule indicates its relative energy: red corresponds to the lowest energy configuration followed by white and blue, respectively.

In order to further clarify the effect of the interactions between hydrogen molecules and the ions, a series of DFT calculations were performed. In each one, a geometry optimization of a system consisting of a hydrogen molecule and an IL ion was carried out. For each H_2_/ion combination, several starting configurations were sampled. The stable final configurations that correspond to the most favorable binding energies can be seen superposed in Figure 4. Colors of the different hydrogen molecules allude to the relative energy of the different configurations, following the red–white–blue color scale from the most stable to the less favored ones.

For , the preferred configurations correspond to the hydrogen molecule directly facing one of the C–N bonds, at a distance of around 3 Å from the edge nitrogen. However, for the  anion, which shares its tetrahedral nature with , the most favorable configurations for hydrogen are offset from the anion symmetry axes. A similar behavior can be observed in the  anion. In both anions, the distance from the H_2_ molecule to the closest ion atom is around 2.5 Å. This difference in orientation is consistent with the disposition of the lone electron pairs of these three ions. Regarding the nonsymmetrical ions, for [EA]^+^, the only stable configuration found corresponds to the hydrogen molecule in front of the apolar tail, at a distance of 3 Å. For [HEIM]^+^, the most favorable positions of H_2_ are more homogeneously distributed around the molecule. In the case of [TFSI]^−^, in the most stable configuration, the hydrogen molecule accommodates at a distance of around 2.6 Å from the nitrogen atom, directly facing it. On the other hand, the  series follows a clear pattern, where all the stable configurations converge to the same location near the imidazolium ring, once again at a distance of around 3 Å to the nearest atom. In particular, the minimum energy conformation is obtained when H_2_ is placed on top of the imidazolium ring, whereas the position corresponding to the third lowest conformational energy is observed to be near the tail. In the gas phase, these would be the most favorable configurations around the IL ions, but in the bulk mixture, the interactions between counterions must be taken into account.

In Table 2, we provide the differences between the binding energies of the most stable final configurations. The binding energy (Δ*E*), defined as the energy required to form a dimer in a given configuration starting from infinitely separated entities [Δ*E* = *E*_H_2_/ion_ – (*E*_H_2__ + *E*_ion_)], is important to determine gas solubility. Note that these binding energies have been calculated using the counterpoise method, as explained in ref (84). As it can be seen, the  anion possesses the largest binding energy. The binding energy is almost constant in the  series, as it was already alluded by the similarities between their stable configurations. Focusing on the energetic differences with respect to the most stable one (Δ*E*_lowest_ = Δ*E* – Δ*E*_1st_), we can observe that for those ion–H_2_ systems in which the third configuration is not equivalent to the most stable ones, this conformation is noticeably less favorable. Nevertheless, all of the binding energies obtained are orders of magnitude below the IL anion–cation pair binding energies, which are shown in Table 3. These larger interactions will take preference over the H_2_–ion interactions that are relegated to a secondary position. Hence, interionic interactions are strong enough to preserve the IL structure upon the addition of H_2_, which confirms our previous MD analysis.

**Table 2:** Binding Energies (Δ*E*) of the Three Most Stable Configurations around Each Molecule and Energy Gap to the Lowest Energy Conformation (Δ*E*_lowest_)

	level	Δ*E* (kcal/mol)	Δ*E*_lowest_ (kcal/mol)
B(CN)_4_	1st	–1.00	
	2nd	–1.00	1.23 × 10^–^^4^
	3rd	–0.80	2.02 × 10^–^^1^
BF_4_	1st	–1.69	
	2nd	–1.69	1.32 × 10^–^^8^
	3rd	–1.69	4.83 × 10^–^^6^
HEIM	1st	–1.72	
	2nd	–1.72	2.14 × 10^–^^5^
	3rd	–1.72	2.15 × 10^–^^5^
EA	1st	–0.49	
	2nd		
	3rd		
NO_3_	1st	–2.36	
	2nd	–2.36	5.84 × 10^–^^8^
	3rd	–2.36	1.14 × 10^–^^7^
TFSI	1st	–1.29	
	2nd	–1.29	2.06 × 10^–^^5^
	3rd	–1.24	5.47 × 10^–^^2^
C_2_MIM	1st	–1.49	
	2nd	–1.49	8.79 × 10^–^^8^
	3rd	–1.05	4.31 × 10^–^^1^
C_4_MIM	1st	–1.61	
	2nd	–1.61	3.37 × 10^–^^6^
	3rd	–1.48	1.31 × 10^–^^1^
C_6_MIM	1st	–1.61	
	2nd	–1.61	1.82 × 10^–^^4^
	3rd	–1.47	1.37 × 10^–^^1^
C_8_MIM	1st	–1.61	
	2nd	–1.61	2.49 × 10^–^^5^
	3rd	–1.47	1.37 × 10^–^^1^
C_10_MIM	1st	–1.61	
	2nd	–1.61	1.58 × 10^–^^4^
	3rd	–1.28	3.29 × 10^–^^1^
C_12_MIM	1st	–1.61	
	2nd	–1.61	5.53 × 10^–^^5^
	3rd	–1.47	1.36 × 10^–^^1^

**Table 3:** Binding Energies (Δ*E*) of the Anion–Cation IL Pairs

pair	Δ*E* (kcal/mol)	pair	Δ*E* (kcal/mol)
[EA][NO_3_]	–120.46	[HEIM][NO_3_]	–85.04
[HEIM][TFSI]	–86.02	[EMIM][B(CN)_4_]	–75.07
[EMIM][TFSI]	–82.34	[EMIM][NO_3_]	–82.53
[C_2_MIM][BF_4_]	–78.69	[C_4_MIM][BF_4_]	–88.39
[C_6_MIM][BF_4_]	–79.96	[C_8_MIM][BF_4_]	–87.04
[C_10_MIM][BF_4_]	–88.05	[C_12_MIM][BF_4_]	–86.94

**Figure 5:**
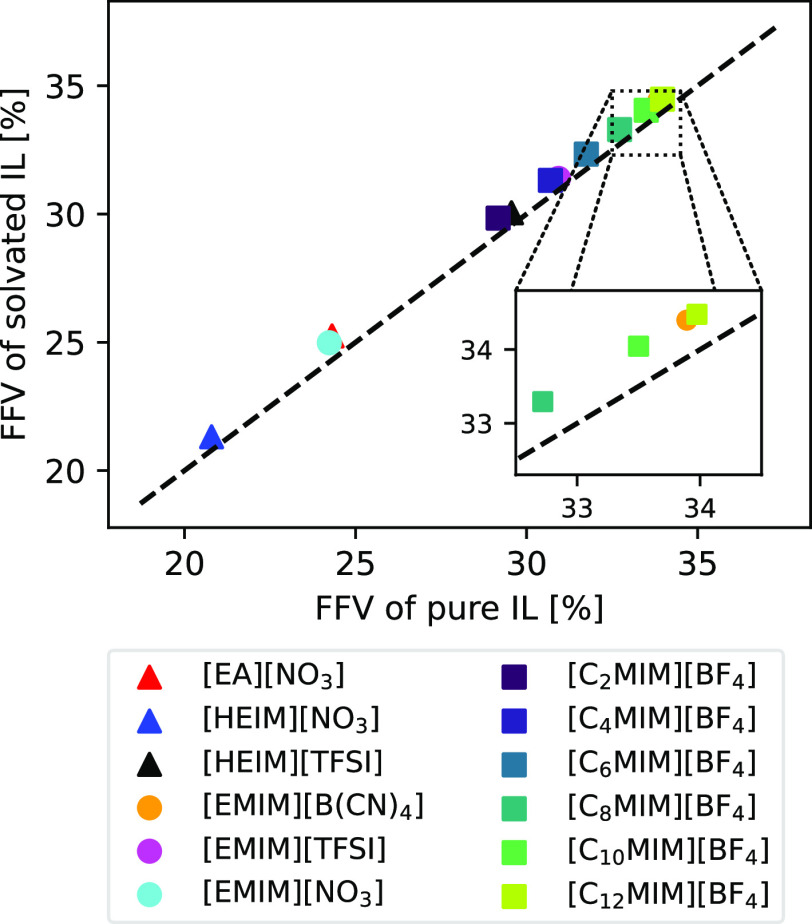
Correlation between the FFV left by the ILs of the systems containing hydrogen and the FFV of the pure ILs. The dashed line corresponds to FFV_H_2__ = FFV_Pure_. Triangles represent protic ILs, circles represent aprotic ILs, and squares represent the C_*n*_MIM series.

As has been shown, the interaction forces between H_2_ and the IL species are not strong enough to influence the structure of the mixture. Therefore, the solubility and accommodation of H_2_ in the mixture will be strongly related to the free space that the cation–anion network leaves unoccupied. To analyze this behavior, the fractional free volume (FFV) of all systems, both with and without molecular H_2_, was computed as
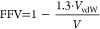
4where *V*_vdW_ stands for the molecular van der Waals volume and *V* is the total volume of the simulation box. The factor of 1.3 accounts for the low temperature limit of the molecular volume^85^ and is widely used in the available literature. Molecular van der Waals volumes were computed using the GROMACS tool with a probe of radius 0. van der Waals radii for the calculations were extracted from refs (86) and (87). Only the FFV left by the ILs was computed (i.e., H_2_ molecules were regarded as an empty space). The results are shown in Figure 5. From the figure, it is apparent that the FFV of the systems does not remarkably change with the addition of H_2_ molecules, showing that gas molecules at a 5 mol % concentration tend to occupy the vacancies left by the IL moieties. Nevertheless, a slight increase in FFV can be seen when H_2_ molecules are introduced, which could indicate that gas molecules force IL ions slightly apart but without significantly changing the structure of the liquid. Overall, the fact that the structure of the systems is not perturbed when H_2_ molecules are added serves as a posteriori evidence that these systems are indeed capable of storing a 5 mol % concentration of hydrogen.

As for the numerical values, it can be seen that the FFVs range between 20 and 35%. These are slightly higher than the previously reported ones,^88−91^ which can be attributed both to the size of some  cations and the high pressure and temperature conditions under which the simulations were carried out. Overall, it can be seen that FFV increases with both cation and anion size, reflecting that bulky components tend to leave more empty space in the fluid since they cannot be tightly packed. Interestingly, the FFV for [C_*n*_MIM][BF_4_] does not increase at a steady rate as the alkyl chain is lengthened, but rather the increment in FFV is smaller the larger the carbon tails are. This asymptotic effect has already been reported by Shannon et al.^92^ and is attributed to the fact that as the alkyl chain length tends to higher values, the FFV of the IL should approach that of a polymer.

**Figure 6:**
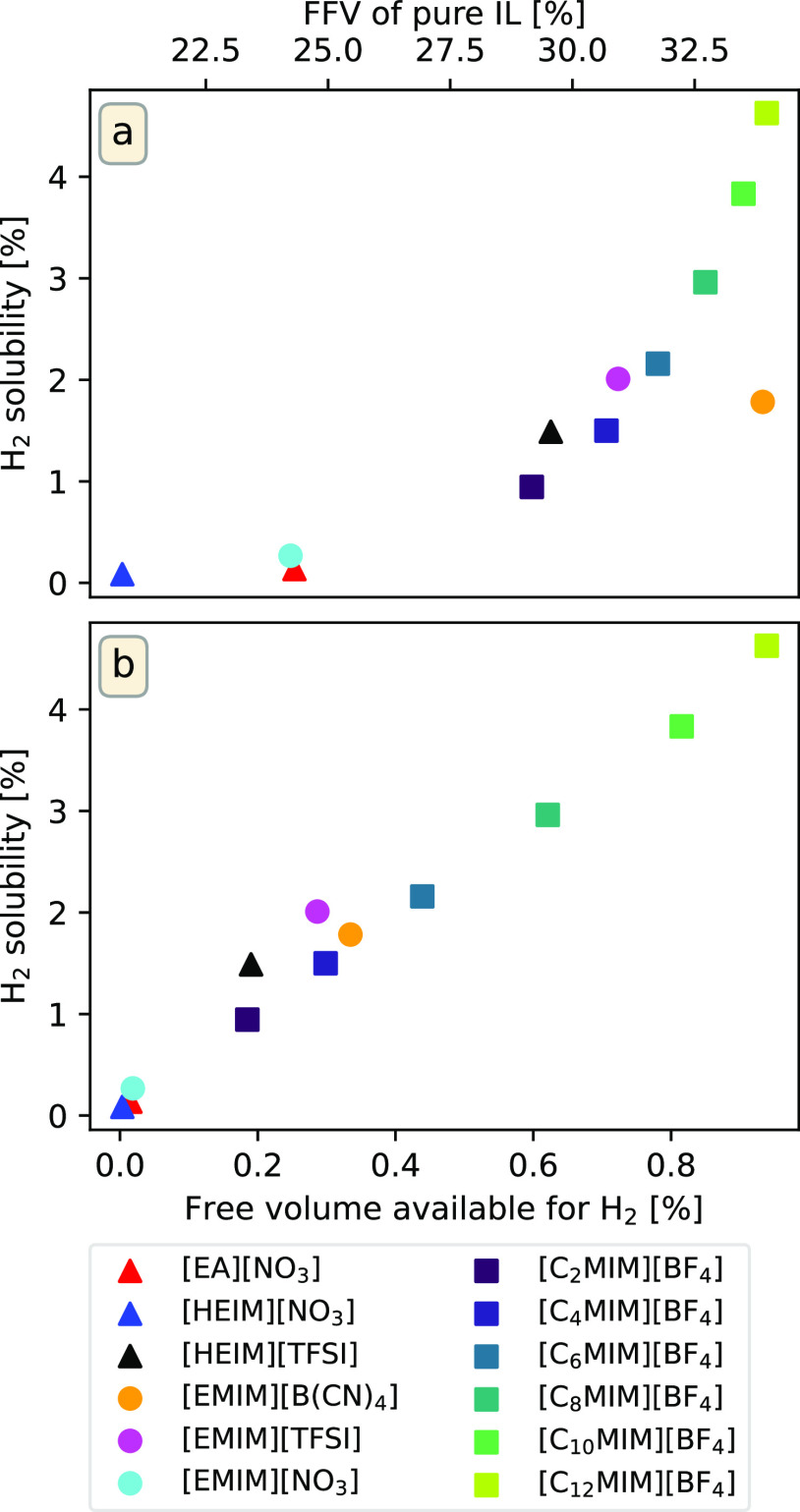
(a) Solubilities of H_2_ molecules in ILs as a function of FFV and (b) free volume available for H_2_ molecules to occupy. Triangles represent protic ILs, circles represent aprotic ILs, and squares represent the C_*n*_MIM series.

Regarding hydrogen solubility, it is possible to correlate the already calculated values (Table 1) with the FFV within the systems. Such dependency is shown in Figure 6a where, for example, a tendency toward greater hydrogen solubility in ILs with longer alkyl chains can be observed. It is clear that solubilization capacity increases with FFV, again showing that the driving factor behind the absorption of H_2_ molecules within ILs is the available free volume inside these systems. This key role of the size and flexibility of the IL ions in determining the solubility of gases has been reported to be characteristic of systems in which there are no strong IL/gas interactions,^32,34,47^ in contrast to heavier gases for which the segregation level of the IL and the formation of polar and apolar domains must be taken into account. However, even though an overall tendency can be seen, there are outliers such as [EMIM][B(CN)_4_], which has a high FFV but exhibits a relatively low hydrogen solubility.

In order to reconcile this apparently contradictory behavior of [EMIM][B(CN)_4_] with the free volume hypothesis, it is important to realize that the relevant metric is not the total available volume but rather the free volume that can be occupied by H_2_ molecules. It is possible for a liquid to have such a structure that it exhibits a large FFV but whose interstitial voids are not large enough to host hydrogen molecules. In Figure 6b, H_2_ solubility is plotted against the volume available to H_2_ molecules, which was calculated using the same GROMACS tool as for the FFV determination but with a probe radius of 0.152 nm.^93^ In the figure, it can be seen that ILs with similar solubilization capacities also have similar free volumes, something specially clear with [EMIM][B(CN)_4_], which exhibits high FFV; however, most of its interstitial voids cannot accommodate H_2_ molecules, thus resulting in gas solubility values more similar to those of [EMIM][TFSI] or [C_4_MIM][BF_4_]. As such, when considering ILs for hydrogen storage, it is important to tune not only the total FFV but also the location and size of the cavities within the system. As will be shown in the following section, this can be achieved by properly tuning the anion–cation pair.

Since the solvation process seems to be mainly driven by the already available free volume within the pure IL, it is expected that FFV plays a role in the energetics of the process. In order to study that influence, solvation enthalpies were computed for all systems, which for a 5 mol % concentration of hydrogen are given by
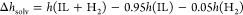
5where all enthalpies are molar and the system for which this quantity was calculated is indicated in parentheses. The quantity *h*(H_2_) was computed from a 10 ns MD simulation of 1000 H_2_ molecules in the NPT ensemble, previously stabilized for 20 ns. All enthalpies were calculated using the GROMACS tools.

**Figure 7:**
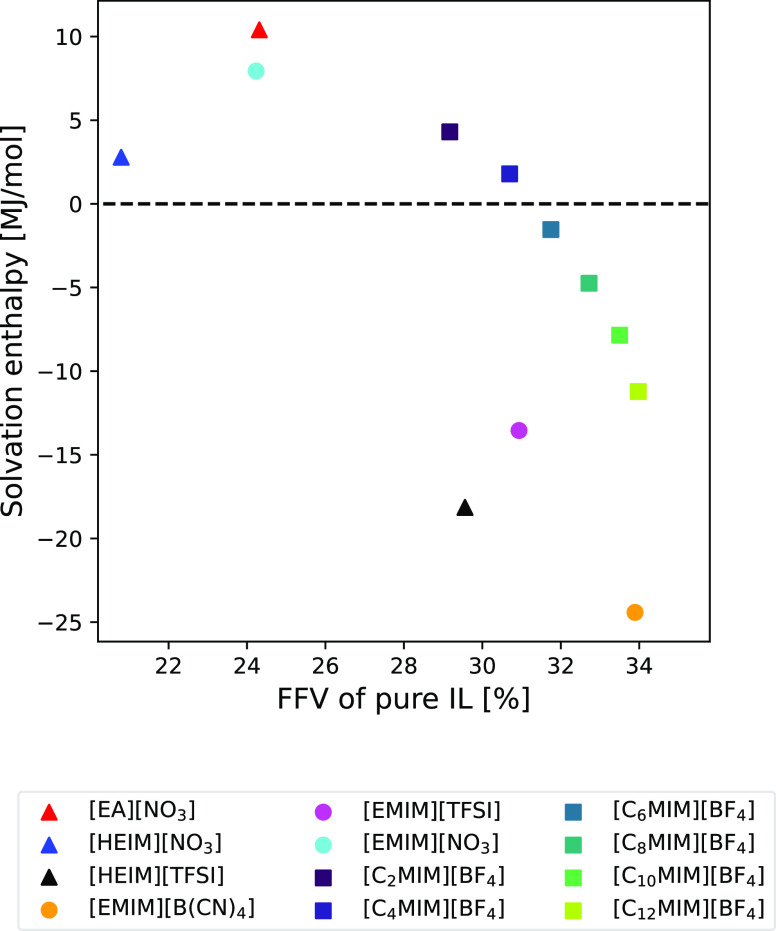
Solvation enthalpy of H_2_ molecules as a function of the FFV of the pure IL obtained from MD simulations for all studied systems. Triangles represent protic ILs, circles represent aprotic ILs, and squares represent the C_*n*_MIM series. The dashed line represents Δ*h*_solv_ = 0.

The results can be seen in Figure 7. Interestingly, the sign of the solvation enthalpy changes depending on the systems, pointing out that the energetics of the solvation process is affected by the particular composition of the IL. In general, Δ*h*_solv_ tends to decrease as the FFV increases. Some of the ILs that present the highest solvation enthalpies are [EA][NO_3_], [HEIM][NO_3_], and [EMIM][NO_3_], all of them sharing the same anion and exhibiting a low FFV, which will be further discussed later. As for the high values obtained for the solvation enthalpies, it has been previously reported in ref (94) that while MD simulations can accurately predict the sign of solvation enthalpies, there exist non-negligible differences between experimental results and simulation data. The values of the solvation enthalpies are frequently overestimated by simulations. In any case, it can be concluded that an increase in the FFV corresponds to a decrease in the solvation enthalpy, meaning that the solvation process is more exothermic when more space is available to the solute. Conversely, the removal of solvated H_2_ molecules from the IL requires less energy the more tightly packed the systems are. Additionally, if not enough volume is available to the solute within the IL, then the solvation process becomes endothermic.

**Figure 8:**
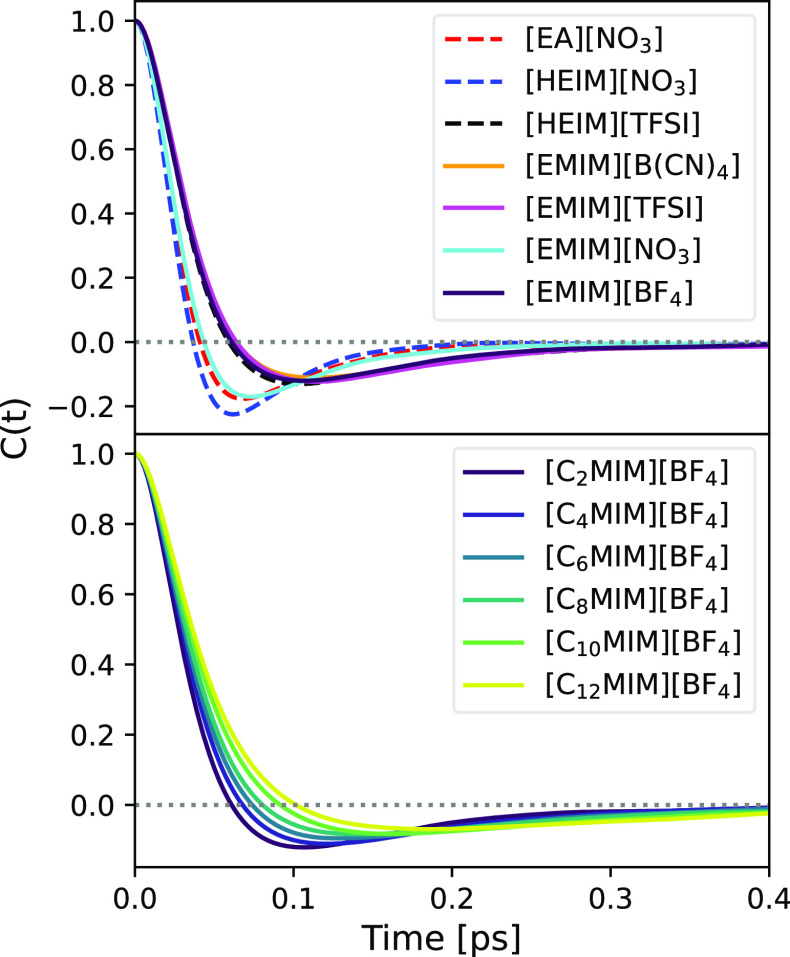
Normalized VACF of H_2_ molecules for different systems. Solid lines represent aprotic ILs, while dashed lines indicate protic ILs. A gray dotted line was included to mark *y* = 0.

Finally, in order to study the dynamics of solvated H_2_ molecules, VACFs of their centers of mass were computed. These functions are given by
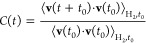
6where  represent the averages over all H_2_ molecules and over all possible time origins. These functions are depicted in Figure 8 for both the main ILs (top) and the C_*n*_MIM series (bottom). For the main ILs, two different behaviors are immediately noticed, one where a larger decrease in the VACF happens at about 50 fs and a smoother one, where the oscillation is damped and the VACF slowly decays to zero at large times.

The VACFs that vanish more rapidly correspond to [EMIM][NO_3_], [HEIM][NO_3_], and [EA][NO_3_], and these ILs also reach the diffusive regime slightly earlier. It is clear from Figure 8 that the influence of the cation over the VACFs is limited since protic and aprotic liquids seem to exhibit both behaviors indistinctly. The difference between the two curves is the presence of the  anion, which leads to a shorter collision time. This hypothesis of cations having little impact is reinforced by the lower plot in Figure 8, where VACFs for the C_*n*_MIM series are shown. It can be seen that as the alkyl chain length of the cation increases, so does the collision time, and the VACF becomes smoother with a less pronounced minimum. Since for large carbon tails, H_2_ molecules are mainly located in the apolar domains due to solvophobic solvation, it is reasonable to identify large collision times with the dynamics that H_2_ molecules experience when located in those regions.

Taking into account that it is unlikely that  anions specifically alter the dynamics of H_2_ molecules in apolar domains, a more plausible explanation is that the presence of  enables H_2_ molecules to access other regions of the mixture, where they experience different dynamics, thus causing a change in the VACFs. We will further explore this hypothesis and the mechanism behind it in the following section.

### Influence of the Anion

It is apparent that while all studied ILs show a tendency of H_2_ molecules to be accommodated in the empty space within apolar domains, the role of the anion cannot be neglected. It has been shown that the choice of anion has a significant influence on the VACFs. Moreover, in Figures 5 and 7, it can be seen how the liquids with  anions tend to have both a low FFV and high solvation enthalpies, thus leading to low hydrogen solubility.

To further analyze the effect of the anions without the presence of different cations influencing the results, the only systems considered for the following analysis were those with [EMIM]^+^ cations. Cumulative RDFs (CRDFs) and RDFs between the carbon of the methyl group (CT) in the [EMIM]^+^ cation ring and both H_2_ molecules and a representative atom from each anion were calculated from IL + H_2_ trajectories. The CRDFs can be obtained from RDFs through the expression
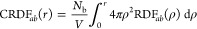
7

**Figure 9:**
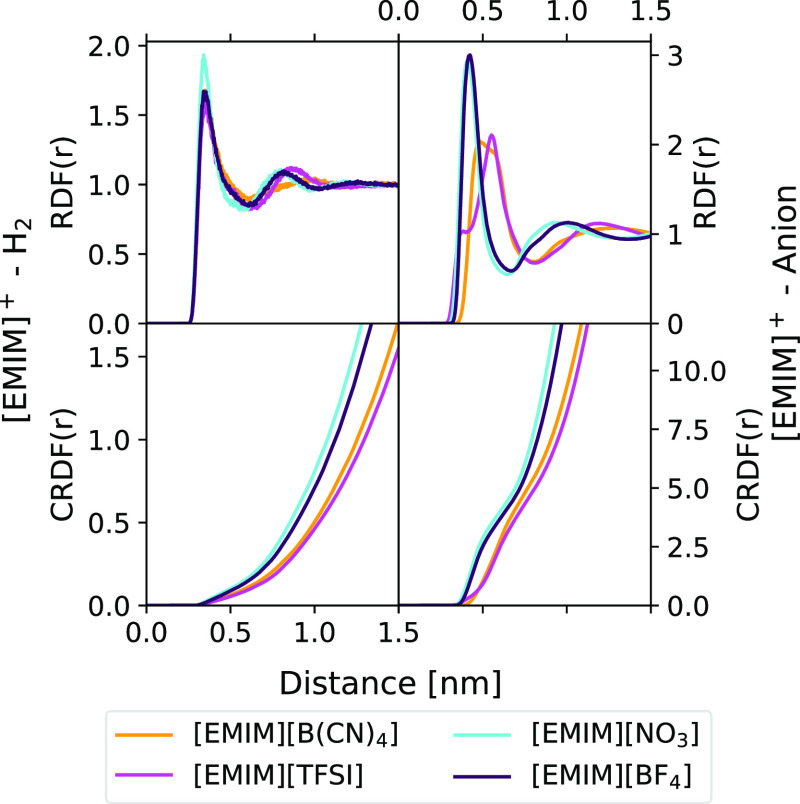
RDFs (top) and CRDFs (bottom) between the CT carbon atom of [EMIM]^+^ cations and the center of mass of H_2_ molecules (left) and between the CT carbon atom of [EMIM]^+^ cations and B, NY, N15, and BF atoms of , [TFSI]^−^, , and  anions, respectively (right).

The value of the CRDF at the distance where the RDF presents its first minimum is the coordination number (CN) of the *b* species around *a*. The results are listed in Figure 9. It can be immediately seen that the affinity of H_2_ molecules for the methyl group of the imidazolium ring is stronger when  anions are present, which results in a higher density of gas molecules around those atoms.

Moreover, regarding cation–anion interactions, the RDFs show that  and  behave in a similar way, exhibiting a sharp peak at 0.4 nm, whereas [TFSI]^−^ and  lead to broader distributions. This is due to the significant size difference between those anions, with the larger ones coordinating at a greater distance from the carbon atom. However, in spite of the changes in the coordination distances, from the CRDFs, it can be seen that the CNs between anions and the methyl group are between 4 and 5 for all anions.

The CNs between H_2_ molecules and the methyl group corroborate the hypothesis that gas molecules can access the polar regions of the IL when the  anion is present, although limitedly. The CRDFs for H_2_ molecules around the methyl group show that their presence is more abundant when the  anion is present. In the case of the other small anion, , the presence of gas molecules is higher than in the case of the large [TFSI]^−^ and  anions, but it is never as high as in the presence of nitrate. The features seen in Figure 9 are compatible with the observations made by Neumann and Stassen^95^ when they concluded that anions with small size and multiple coordination sites are desired for coordinating with gas molecules such as N_2_.

This larger concentration of H_2_ molecules near the methyl group of the imidazolium ring when  is present can be explained on the basis of free volume. As previously mentioned, approximately 4 or 5 anions are coordinated around the carbon atom, and if the said anions are big, they will occupy most of the available volume in that region and thus prevent H_2_ molecules from being located in that area. The fact that, out of the four anions,  is both small in size and has a planar geometry forces it to leave the most free volume near the carbon heads. Both [TFSI]^−^ and  are significantly bigger, while the tetrahedral geometry of  restricts more volume than a planar one, specially when four anions are coordinated near each other.

**Figure 10:**
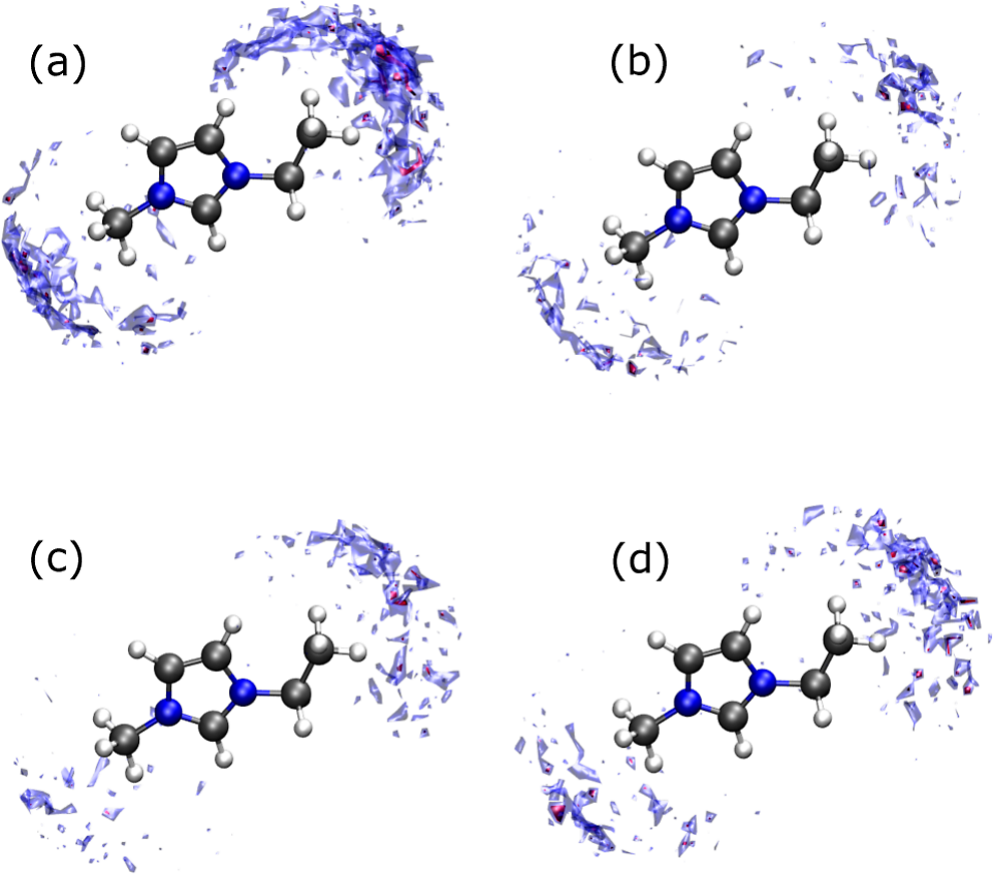
Spatial distribution functions of H_2_ molecules around IL cations for different anion choices: (a) , (b) , (c) , and (d) [TFSI]^−^. Blue regions indicate a density of H_2_ molecules equal to 3.5 times that of the bulk, whereas red regions show zones where the said density is 4.5 times the bulk density of hydrogen in the mixture.

This effect can be clearly seen in Figure 10, where the spatial distribution of hydrogen molecules around the IL cations is displayed. From the figure, the influence of the  anion is clear. When it is present, the density of H_2_ molecules around the methyl group attached to the polar head of the cations is much higher than that for the other anions. Then, it is clear now that the two dynamic regimes shown in the VACFs of Figure 8 correspond either to the dynamics of H_2_ molecules situated in apolar regions composed of alkyl chains or, in -based ILs, to gas molecules also situated in polar regions where both cations and anions are present, if the size and coordination layout allow for enough free volume to be formed. Therefore, it is clear that both the size and geometry of the anions, together with their way of coordinating themselves around cations, play an important role in the solvation process, since it is a source of free volume, the driving force behind H_2_ solvation in ILs. In this case, our observations suggest that -based ILs are more tightly packed and leave overall a smaller amount of free volume to accommodate hydrogen molecules; these voids being distributed both in the polar and in the apolar domains of the IL. However, the other ILs have a greater amount of free volume that is mainly located in the apolar regions. This also provides evidence that a choice of the ions that facilitates light gases such as H_2_ to be more homogeneously distributed in space does not translate into better solubility, but instead, it is the probability of hydrogen-sized cavity formation that must be considered.

## Conclusions

In this work, the underlying mechanisms governing the solvation of molecular hydrogen in several ILs were examined by means of MD simulations at *T* = 550 K and *p* = 50 bar. The hydrogen mole fraction was set at 0.05 for all the systems after analyzing solubility using Widom’s particle insertion technique. Additional DFT simulations were carried out in order to get a deeper insight into the preferential interaction sites for this gas in IL cations and anions.

Through the analysis of the RDFs, we observed a weak coordination between hydrogen molecules and the ions. In addition, hydrogen was mainly dissolved in the apolar regions of the systems, except for those ILs containing nitrate, in which the gas was also accommodated in the ionic domains. These two different arrangements were clearly reflected in the two dynamic regimes shown by the VACFs. Calculation of the FFV further reveals that the addition of gas molecules does not remarkably modify the nanostructure of pure ILs or their properties, but they are instead absorbed into the already existing cavities. Thus, our study provides strong evidence of the free volume available for hydrogen intake as the main effect dictating the solubility of this gas in ILs.

Having the knowledge for successfully tuning the factors that affect the solubility of gases in ILs is of fundamental importance for different specific applications, such as separation processes. Our findings suggest that the choice of the ions comprising the ILs must be made by focusing on the easing of cavity formation for increasing the available free volume, for example, by means of cations with long chain lengths or anions with a flexible structure. Conversely, if low hydrogen absorption is desired, ILs composed of densely packed cations and anions are preferable. For example, those based on the nitrate anion can be considered as good candidates, since our results show that even though the solvation process seems to be the most favorable one from the energetic point of view, the available voids within the ionic network can accommodate a very low amount of hydrogen, thus resulting in low gas solubility. This reveals that interactions other than excluded volume play a secondary role in the solvation mechanism of hydrogen.

In conclusion, this study serves as another proof of the high customizability presented by ILs, which have been shown to display a wide range of behaviors as solvents for molecular hydrogen. The results obtained are, however, limited to the bulk regime of the ILs, neglecting any possible interfaces. The effect of nanoporous confinement of the ILs on their absorption capacity will be explored in future work.

## Data Availability

DFT simulations were performed with Gaussian 16, revision C.01.^69^ Initial configurations for DFT simulations were generated with Avogadro (https://avogadro.cc/).^96^ MD simulations were performed with version 2020.4 of GROMACS.^54^ Initial simulation boxes for MD were created with PACKMOL (https://m3g.github.io/packmol/).^65^ MD trajectories were evaluated, and figures were drawn using the MDAnalysis (https://www.mdanalysis.org/),^97,98^ Matplotlib (https://matplotlib.org/),^99^ SciPy (https://scipy.org/),^100^ and NumPy (https://numpy.org/)^101^ libraries. MDDFs were computed using Julia (https://julialang.org/),^102^ with the ComplexMixtures.jl module (https://github.com/m3g/ComplexMixtures.jl).^80^ 3D models of the molecules were done with MolView (https://molview.org/).^103^ Spatial distribution functions were represented with Visual Molecular Dynamics (https://www.ks.uiuc.edu/Research/vmd/).^104^ Table of contents was rendered using Adobe Photoshop 2020.^105^ Input files and analysis scripts are available at 10.5281/zenodo.8112674.

## Supporting Information Available

Computational calculations of the excess chemical potential and the minimum distance distribution functions between the ions and hydrogen molecules (PDF)
