# Supplementary material for: Size Matters: A Computational Study of Hydrogen Absorption in Ionic Liquids
Source: J Chem Inf Model. 2023 Dec 21;64(1):164–77. doi: 10.1021/acs.jcim.3c01688 (PMC10777413; doi:10.1021/acs.jcim.3c01688)
Supplement: Supplementary file 1 — ci3c01688_si_001.pdf [file ci3c01688_si_001.pdf]

Supporting information

Size Matters: A computational study of hydrogen absorption in ionic liquids

Alejandro Rivera-Pousa,[†,‡,§] Raúl Lois-Cuns,[†,‡,§] Martín Otero-Lema,[†,‡,§] Hadrián Montes-Campos,[†,‡,¶] Trinidad Méndez-Morales,[*,†,‡] and Luis Miguel Varela[*,†,‡]

†*Grupo de Nanomateriais, Fotónica e Materia Branda, Departamento de Física de Partículas, Universidade de Santiago de Compostela, Campus Vida s/n, E-15782, Santiago de Compostela, Spain*

‡*Instituto de Materiais (iMATUS), Universidade de Santiago de Compostela, Avenida do Mestre Mateo 25, E-15782, Santiago de Compostela, Spain*

¶*CIQUP, Institute of Molecular Sciences (IMS)—Departamento de Química e Bioquímica, Faculdade de Ciências da Universidade do Porto, Rua Campo Alegre, 4169-007, Porto, Portugal*

§*Contributed equally to this work*

E-mail: trinidad.mendez@usc.es; luismiguel.varela@usc.es

1 Excess chemical potential

Here we represent the excess chemical potential as a function of simulation time during the calculation of solubility following Widom's particle insertion method. It can be seen that in all cases, the average chemical potential oscillates around its equilibrium value.

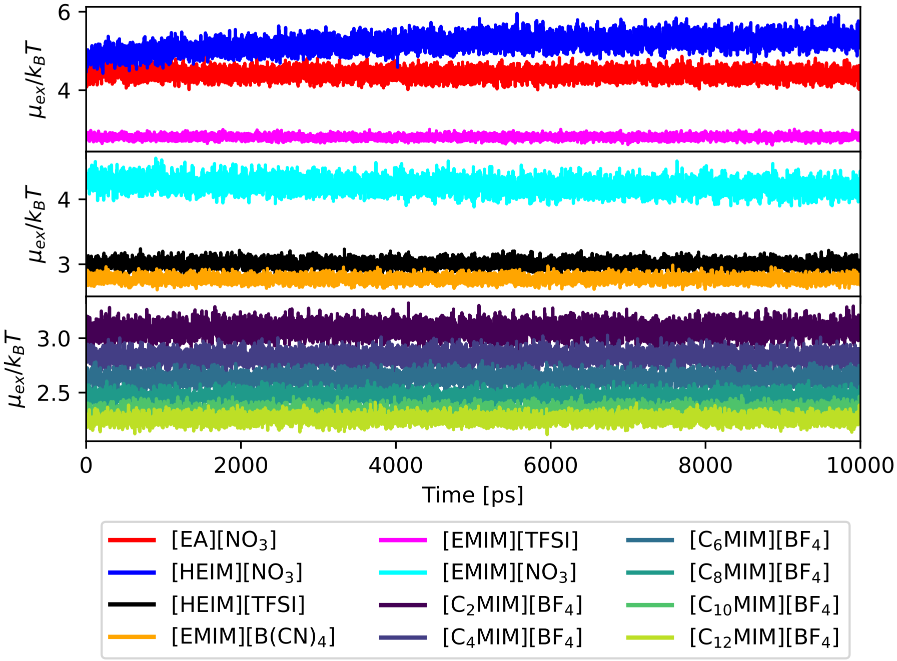

Figure S1: Excess chemical potential as a function of simulation time.

2 Minimum distance distribution functions

For a system composed of a solvent and a solute, the minimum distance distribution function (MDDF) between solvent molecules and the solute is the distribution function of the minimum distance between any solute atom and any atom belonging to the solvent molecules of interest. Here we represent the MDDFs between hydrogen molecules (solute) and cations/anions for every IL (solvent) in our study.

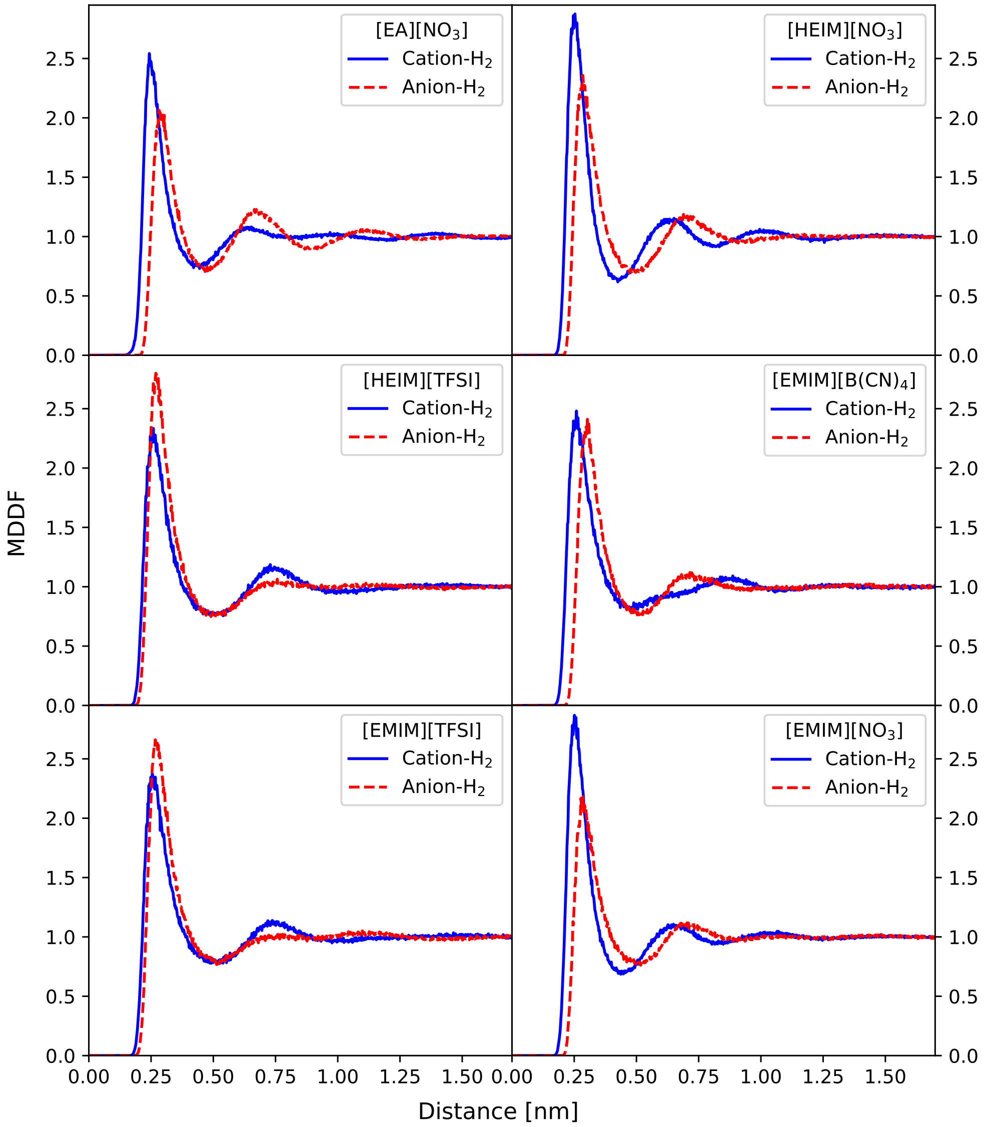

Figure S2: MDDFs between cations/anions (solvent) and H_2 molecules (solute).

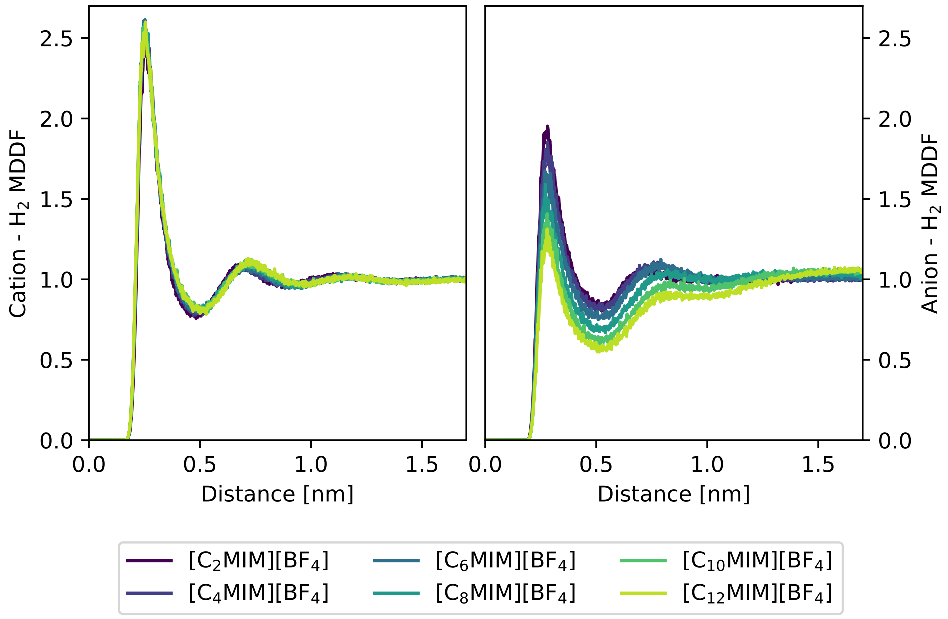

Figure S3: MDDFs between cations/anions (solvent) and H_2 molecules (solute) for the [C_nMIM][BF_4] series.
